# The relationship between childhood adversity, recent stressors, and depression in college students attending a South African university

**DOI:** 10.1186/s12888-017-1583-9

**Published:** 2018-03-09

**Authors:** Sumaya Mall, Philippe Mortier, Lian Taljaard, Janine Roos, Dan J. Stein, Christine Lochner

**Affiliations:** 1 Department of Psychiatry and Mental Health, University of Cape Town, Cape Town, South Africa; 2 Division of Epidemiology and Biostatistics, School of Public Health, University of the Witwatersrand, Johannesburg, South Africa; 3 Research Group Psychiatry, Department of Neurosciences, KU Leuven, Belgium; 4 MRC Unit on Risk and Resilience in Mental Disorders, Department of Psychiatry, Stellenbosch University, Stellenbosch, South Africa; 5 Mental Health Information Centre of Southern Africa, Stellenbosch, South Africa

**Keywords:** College students, Depression, Childhood adversity, Recent Stressors, South Africa

## Abstract

**Background:**

College students are at risk of depression. This risk may be increased by the experience of childhood adversity and/or recent stressors. This study examined the association between reported experiences of childhood adversity, recent stressors and depression during the last 12 months in a cohort of South African university students.

**Methods:**

Six hundred and eighty-six first year students at Stellenbosch University in South Africa completed a health-focused e-survey that included items on childhood adversity, recent stressors and mood. Individual and population attributable risk proportions (PARP) between experiences of childhood adversity and 12-month stressful experiences and 12-month depression were estimated using multivariate binomial logistic regression analysis.

**Results:**

About one in six students reported depression during the last 12 months. Being a victim of bullying and emotional abuse or emotional neglect during childhood were the strongest predictors of depression in the past year at both individual and population level. With regard to recent stressors, a romantic partner being unfaithful, serious ongoing arguments or break-ups with some other close friend or family member and a sexual or gender identity crisis were the strongest predictors of depression. The predictor effect of recent stressors was significantly reduced in the final model that adjusted for the type and number of childhood traumatic experiences. At a population level, academic stress, serious ongoing arguments or break-ups with a close friend or family member, and serious betrayal by someone close were the variables that yielded the highest PARP.

**Conclusions:**

Our findings suggest a significant relationship between early adversity, recent stressors, and depression here and throughout, consistent with the broader literature on predictors of depression. This study contributes to the limited data on college students’ mental health in low and middle income countries including on the African continent. The findings provide information on the population level effect sizes of trauma as a risk factor for depression, as well as on the relationship between specific recent stressors and depression in college students.

**Electronic supplementary material:**

The online version of this article (10.1186/s12888-017-1583-9) contains supplementary material, which is available to authorized users.

## Background

Several studies suggest that exposure to early childhood adversity or recent stressors (i.e. experiences during adulthood) are associated with increased risk of depression during adolescence or adulthood [1–4]. Not only could specific experiences of such adversity during childhood (e.g. physical abuse) increase the risk of depression in adulthood more significantly than others [5–8], but repeated trauma exposure may result in more severe symptoms of psychopathology [9, 10]. Further, research has suggested that cumulative stressful experiences, i.e. experience of childhood adversity and recent stressors may interact to increase the risk of depression in adulthood [11, 12].

Although research suggests that depression is common among college students [13, 14] and that childhood adversity may be associated with psychopathology (e.g. depression or alcoholism) in this group [15, 16], several gaps remain in the field. First, a recent meta-analysis of mental health interventions for college students suggests that previous studies of this group have been restricted to higher income countries suggesting a need to conduct studies in low and middle income countries including on the African continent [17]. Second, to our knowledge, no studies have examined population level effect sizes of trauma as risk factor of depression in college students. These population effects can be calculated with population attributable risk proportions (PARP). Assuming a causal relationship between risk factors and outcome, PARPs provide an estimate of the potential reduction in depression prevalence should a particular risk factor be removed from the population [18, 19]. They take into account that highly prevalent risk factors carrying low individual-level risk may be as important to consider as low prevalent risk factors carrying high risk for the affected individuals. Third, while previous studies suggest that a summary score of recent stressors may well be predictive of depression, particularly among adolescents, there is less research on whether specific recent stressors are stronger predictors of depression than others [20, 21].

Here we address these gaps in the literature in a study of a cohort of undergraduate students in South Africa. We hypothesized that in this group, 1) there would be a positive association between depression (i.e. in the last 12 months) and the number of childhood adverse and recent stressors experienced, 2) that different types of childhood trauma and recent stressors may be more significant in terms of their role in depression than others, 3) having any childhood adversity or any recent stressor has a significant role in predicting depression (i.e. in the last 12 months). Finally, we also aimed to calculate PARP, the rationale for which has been provided above [22].

## Methods

### Aims

This study, conducted at one university in South Africa, is embedded in an international, multi-site investigation into the wellness of undergraduate students known as the World Health Organization (WHO) World Mental Health Initiative Surveys International College Student Project (WMH-ICS). This parent study is an epidemiological study that aims to determine the prevalence and correlates of psychiatric disorders among college students in several participating nations. The WMH-ICS also aims to investigate and highlight paths to mental healthcare undertaken by students, and to encourage and promote treatment-seeking behavior [23].

### Study design, setting and participants

The study was conducted at Stellenbosch University (SU), a public research university 40 km from Cape Town. In 2015, when data collection took place, more than half the students were white and females.

The data presented are cross-sectional data. All first year students who entered SU for the first time in 2015 received an email in March of that year, inviting them to participate in this study. A link to the e-survey with questions about lifetime history of risk and protective factors for negative outcomes, such as academic, mental and physical health problems (including past experience of those outcomes) were included. Students received monthly reminders of the survey between March 2015 and November 2015. Data collection ceased in November 2015.

Only students aged 18 or older, who provided informed consent (see below for more details), were allowed to progress into the survey.

All first year students at SU in 2015 (*n* = 5338) were invited to participate in this study. Our sample consisted of 686 participants (12.9% of the original invited sample). Of these 377 were female (54.9%) and 309 were male (45.1%). With regard to ancestry, 66.3% of participating students were white, 18.9% ‘coloured’^1^, 12.1% were black and 2.7% of Indian descent (See Table 1 below for percentages weighted by gender and race).

**Table 1:** Sociodemographic characteristics of the sample

Covariate	n(w)^a^	%(w)^a^	SE
Gender			
Male	309	45.1	1.9
Female	377	54.9	1.9
Race			
White	455	66.3	1.7
Coloured	129	18.9	1.5
Black	83	12.1	1.1
Indian	18	2.7	0.5

^a^weighted for gender and race

### Materials and measures

Several measures were employed in the survey. Those relating specifically to trauma, recent stressors and depression are described below:

#### Childhood adversity

Selected items from validated instruments including: the CIDI-3.0 childhood section [24], The Adverse Childhood Experiences International Questionnaire (ACE-IQ) [25] and the Bully Survey [26], were employed to measure adverse experiences prior to the age of 17. The domains that were extracted from the (ACE-IQ) explore several different experiences of childhood adversity including a) parental psychopathology (e.g. *‘One of your parents (or the people who raised you) had a serious emotional or mental health problem’*, b) intimate partner violence during adolescence (e.g. *How often were you in a romantic relationship where your partner repeatedly said hurtful or insulting things to you?*, c) sexual assault (e.g. *‘You were sexually abused at home’*) and physical assault, (‘e.g. *You were physically abused at home*’) d) emotional abuse (e.g. *You felt loved and cared for by your family*), e) bullying (e.g. *How often were you bullied at school verbally (*i.e.*, teased, called names*)? and f) emotional and physical neglect (e.g. *‘You had to do chores too hard and dangerous for someone your own age’*). For the most part, our measures specifically ask about family-related adversity (e.g. physical assault perpetrated by a family member). Questions about sexual assault are not restricted to assault by family members, but include questions about assault by any perpetrator. All experiences are measured on a Likert scale of 1–5 where 1 indicates ‘never’, 2 indicates ‘rarely’, ‘3 indicates sometimes’ 4 indicates ‘often’ and ‘5’ indicates ‘very often’. For meaningful comparisons between exposed and unexposed groups at both individual level and PARP analysis, the dichotomously coded variables were set at “*rarely*” for all items, except for bullying for which the cut-off value was set at “*sometimes*”. These scores were consistent with previous WMHS studies [27, 28].

#### Recent stressful experiences

Questions about recent stressors were adapted from the Life Events Questionnaire (LEQ) [29], which has been shown to possess sound psychometric properties [30]. The LEQ dichotomously assesses the experience of threatening events that occurred during the previous 12 months. Recent stressors or stressful experiences were defined as the occurrence of a stressful event in the last 12 months. Twenty recent stressors were measured in the questionnaire. These included, but were not limited to, serious illness, death of a friend, romantic relationship difficulties, academic stress, sexual and gender identity crisis, unwanted pregnancy, HIV diagnosis, legal difficulties or being involved in crime.

#### Depression occurring in the past year

We selected three items to measure depression and (from sub-syndromal depression to major depressive disorder) to carefully estimate the burden of this disorder among college students. These criteria are based on the DSM-5 criteria and take into account that depression symptoms lie on a spectrum from symptoms to minor depression to the full disorder [31–34]. This broad concept of depression refers to individuals who have two or more (but less than five) symptoms of depression that have endured for at least two weeks and that are associated with impairment [35]. Respondents were asked to report the experience or frequency of the following symptoms in the past year: depressed mood (‘how often did you feel sad or depressed’), low interest (‘how often did you take little or no interest and pleasure in things’) and interference in their daily activities (‘how often did you feel so low that it interfered with your work or personal life?’) on a Likert scale of 1–5, ranging from “None of the time” (1) to “All or almost all of the time” (5). If they reported two core symptoms and interference in their work or daily lives ‘sometimes’ or more, they were categorized as having ‘broad depression’ [36]. We extracted reports of depression in the past year to associate more closely in time with recent stressful experiences. As described above, the recent stressful experiences had been reported as occurrences during the past year.

### Ethical issues

After permission for conducting the study was obtained from the Institutional Review Board of SU and the University’s Institutional Research and Planning Division, an email using the students’ university email addresses, was sent to all first year students with a description of the study and a link to the e-survey.

In the invitation, students were informed that their participation in the survey was completely voluntary and that their responses would be treated as confidential. They were also informed that the survey results would be anonymous, that there may be potential discomforts (i.e. fatigue, inconvenience) and that there may also be benefits (i.e. contribution to generalizable knowledge). It was also emphasized that the students can withdraw from participation, with no negative consequences. However, due to the anonymity of the survey, they were notified that they will not be able to withdraw from the survey once they had submitted their responses.

Students were also made aware that they will be contacted in subsequent (pre-graduate) years for a follow-up survey.

A phone number to a general study helpdesk was included in the informed consent for students who had questions or concerns. Students who were distressed or requiring urgent assistance were provided with contact details of the 24 h Crisis Line of the local Centre for Student Counselling and Development (CDSC) and the Mental Health Information Centre of Southern Africa. Participants were not able to complete the survey more than once.

Students were not paid to complete the survey. There was however an incentive to compete for a cash prize of 1000 South African rand (approximately 72,8 US dollars).

### Data analysis

All analyses were conducted with SAS (version 9.4).

Nonresponse propensity weighting [37] was employed to account for nonresponse bias (i.e. potential differences in prevalence of depression between survey respondents and non-respondents) [38]. Nonresponse propensity weights were calculated using socio-demographic variables available for the full student population (i.e. race and gender of first year students). A logistic model was developed to predict survey response (vs. nonresponse), with gender and race as predictors. There is little variation in age of first year students so we did not include age as a predictor in these models. Based on this multivariable regression equation, predicted probabilities were calculated. Final non-response propensity weights were obtained by taking the inverse of the predicted probabilities, followed by a normalization procedure to reassure that the sum of the final weights matches the actual sample size. Prevalence estimates are reported as weighted numbers, weighted proportions, and associated standard errors. Logistic regression procedures were used to explore the individual-level associations between predictors (i.e., childhood-adolescent traumatic experiences, and 12-month recent stressors) and the outcome variable (i.e., having depression in the last 12 months). Parameter estimates are reported as odds ratios (OR) and associated 95% confidence intervals. Apart from the specific type of traumatic or stressful experience, we also investigated the association between the presence of *any* experience in each risk domain under study, and 12-month depressive symptoms, as well as the *number* of specific experiences (categorized into having 1, 2, or 3 or more experiences with zero as the reference category) in each risk, and depression over the last 12 months. All analyses were (additionally) adjusted for gender and race as suggested by previous research examining mental health of college students [39, 40]. To estimate population-level risk for 12-month depression, PARP proportions [22] were calculated for each predictor under study, using as a summary predictor the probabilities resulting from the logistic regression equations [41, 42]. PARP provides an estimate of how many outcome cases (i.e., students with depression) are associated with a particular predictor variable under study. As such, assuming a causal relationship between predictor and outcome, PARP can be considered as an estimate of the proportion of 12-month depression that could be potentially alleviated if (the impact of) a particular predictor variable was removed from the population. It should be pointed out, however, that similar to individual-level estimates of risk (OR), PARP estimates in our cross-sectional research design are flawed by the inability to establishing temporality between predictor and outcome, and by the inability to take into account potentially important confounder variables. As with OR, all PARP estimates were adjusted for gender and population group. Finally, a series of multivariable models were estimated. Predictors were entered in blocks, beginning with recent stressors, and followed by childhood-adolescent traumatic experiences, giving us the opportunity to look at the cumulative effect of childhood trauma and recent stressful experiences. Our initial objective was to include all predictor variables under study in the final multivariate models. However, due to estimation problems related to data sparseness, low-prevalent predictor variables (*n* < 30; prevalence <5%) were eliminated. Nagelkerke pseudo-R^2^ was used as a measure of total effect size, and Area-under-the-Curve as measure of prediction accuracy [43]. Firth’s penalized likelihood estimation was consistently applied to avoid inconsistent estimators due to data sparseness [44].

## Results

### Prevalence estimates of socio-demographic variables

The prevalence of depression over the last 12 months was estimated at 16.1% with an associated standard error (SE) of 1.3. Examining the prevalence of adverse events prior to the age of 17 suggested that 79.4% of respondents of the full sample (i.e. with or without depression) reported experiencing at least one childhood adverse event. The three most commonly reported childhood adverse events across the whole sample were bullying (50.0%), emotional abuse (37.2%) and parental psychopathology (47.6%). With regard to recent recent stressors, almost all (90.7%) participants in the whole sample reported experiencing at least one recent stressor. Academic stress was most common, with 78.6% of the students reporting this experience. Other recent stressors, albeit less common, include breaking up with a romantic partner (30.5%) or the death of a close friend or family member (26.8%).

### Bivariate correlates of 12-month depressive symptoms

The following experiences of childhood adversity prior to the age of 17 were significantly associated with depression in the past year: parental psychopathology (OR = 2.1; 95% CI = 1.3–3.2), emotional abuse (OR = 3.2; 95% CI = 2.0–5.0), sexual abuse (OR = 3.0; 95% CI = 1.3–7.1), neglect (OR = 3.4; 95% CI = 1.9–6.0), bullying (OR = 3.0; 95% CI = 1.9–4.8) and dating violence (OR = 1.9; 95% CI = 1.2–3.3). There was a clear dose-response relationship between childhood adversity and depression. Having any adverse experience was associated with a 4.6 increased risk of depression (OR = 4.6; 95% CI = 2.0–10.4) with gender and race adjusted ORs ranging from 4.6 to 8.0 for three or more types of adversity (95% CI = 3.4–18.8). Having an experience of any adversity, three or more adversities and bullying yielded the highest PARP (ranging from 41.3% to 67.9%) (See Table 2).

**Table 2:** Childhood-adolescent adversity as bivariate predictors for the onset of depression during the past year

	prevalence							Chi-square test			Odds Ratio^e^			PARP^e^
Predictor	n(w)	%(w)^a^	SE	%(w)^b^	SE	%(w)^c^	SE	X_2_	df	*P*-value^d^	OR	95%-	95%+	%(w)
Parental psychopathology	308	47.6	1.9	44.7	2.1	60.8	4.4	9.03	1	**0.002**	**2.1**	1.3	3.2	**26.7**
Physical abuse	134	20.4	1.5	19.1	1.7	25.5	4.0	2.22	1	0.139	1.8	1.1	3.1	9.3
Emotional abuse	240	37.2	1.8	31.9	2.0	57.0	4.5	23.63	1	**<0.001**	**3.2**	2.0	5.0	**34.3**
Sexual abuse	28	4.4	0.7	3.3	0.7	9.0	2.4	7.06	1	**0.006**	**3.0**	1.3	7.1	**5.2**
Neglect	84	12.9	1.3	10.6	1.3	22.6	3.8	11.39	1	**0.001**	**3.4**	1.9	6.0	**13.0**
Bullying victimization	324	50.0	1.9	45.2	2.1	70.7	4.1	22.36	1	**<0.001**	**3.0**	1.9	4.8	**41.3**
Dating violence	102	15.7	1.3	13.8	1.4	24.6	3.8	7.75	1	**0.005**	**1.9**	1.2	3.3	**9.8**
Any adverse experience	484	79.4	1.6	76.4	1.8	93.6	2.4	14.52	1	**<0.001**	**4.6**	2.0	10.4	**67.9**
No adverse experience	126	20.6	1.6	23.6	1.8	6.4	2.4	–	–	–	(ref)	(ref)	(ref)	(ref)
Exactly one adverse experience	165	27.1	1.7	28.5	1.9	23.4	3.9	–	–	–	**2.9**	1.2	7.0	**14.1**
Exactly two adverse experiences	126	20.6	1.6	21.2	1.8	18.5	3.6	–	–	–	**3.5**	1.4	9.0	**12.1**
Three or more adverse experiences	194	31.7	1.8	26.7	1.9	51.7	4.6	28.98	3	**<0.001**	**8.0**	3.4	18.8	**42.3**

All OR and PARP estimates are adjusted for gender and race. Significant *P*-values, OR and PARP are indicated in bold (α = 0.05); OR = odds ratio; PARP = population- attributable risk proportion

^a^The prevalence of the predictor in the full sample

^b^The prevalence of the predictor among those without 12-month depressive symptoms

^c^The prevalence of the predictor among those with 12-month depressive symptoms

^d^Unadjusted for multiple comparison. For multiple test adjusted *P*-values, see Additional file 1: Table S1

^e^Adjusted for gender and race

With regard to recent stressors, the following stressors were significantly associated with depression in the past year: discovery that a romantic partner had cheated (OR = 2.4; 95% CI = 1.3–4.3), serious betrayal by someone close to you (OR = 2.1; 95% CI = 1.3–3.4), serious ongoing arguments or breakups with a close friend or family member (OR = 2.5; 95% CI =1.6–4.1], academic stress (OR = 2.1; 95% CI = 1.1–3.9), sexual or gender identity crisis (OR = 3.3; 95% CI = 1.7–6.7) and ‘other’ recent experience (OR = 2.4; 95% CI = 1.5–4.4). After disaggregation for the specific number of stressful experiences, we found evidence for an effect of three or more recent stressor (OR = 3.1; 95% CI = 1.1–8.8). From a population-level perspective, academic stress, three or more stressors and serious ongoing arguments were the variables that yielded the highest PARPs ranging from 18.0–39.6%.

### Multivariable correlates of 12-month depression

Finally, a series of multivariable models were estimated giving us the opportunity to look at childhood adversity and recent stressors cumulatively (See Table 3). A first model found that, when adjusting for both type and number of 12-month recent stressors, three 12-month recent stressors remained significantly associated with depressive symptoms, i.e., a romantic partner being unfaithful, serious ongoing arguments or break-ups with a close friend or family member and a sexual or gender identity crisis, with ORs ranging from 2.1–3.0, and PARPs ranging from 6.9–13.0%. The effect of 12-month stressful events became non-significant in the final model (Nagelkerke pseudo-R^2^ = 15.0%; AUC = 0.79) that additionally adjusted for type and number of childhood traumatic experiences, though it is worth noting that the OR decrease was minimal (see Table 4).

**Table 3:** Recent stressors as bivariate predictors for the onset of depression during the past year, adjusted for gender and race

	prevalence							Chi-square test			Odds Ratio^e^			PARP^e^
Predictor	n(w)	%(w)^a^	SE	%(w)^b^	SE	%(w)^c^	SE	X_2_	df	*P*-value^d^	OR	95%-	95%+	%(w)
A life-threatening illness or injury of a very close friend or family member	161	24.2	1.6	24.7	1.8	22.5	3.7	0.24	1	0.622	0.8	0.5	1.4	−3.3
Death of a close friend or family member	179	26.8	1.6	28.1	1.9	21.5	3.7	1.89	1	0.165	0.7	0.4	1.2	−6.2
Break-up with a romantic partner	202	30.5	1.7	29.0	1.9	37.7	4.4	3.11	1	0.074	1.5	1.0	2.3	10.1
You discovered that a romantic partner cheated on you	73	11.0	1.2	9.6	1.2	19.7	3.6	8.97	1	**0.003**	**2.4**	1.3	4.3	**9.1**
Serious betrayal by someone else close to you	141	21.2	1.5	18.7	1.6	34.7	4.2	13.43	1	**<0.001**	**2.1**	1.3	3.4	**15.1**
Serious ongoing arguments or break-ups with some other close friend or family member	136	20.6	1.5	17.1	1.6	35.2	4.3	17.85	1	**<0.001**	**2.5**	1.6	4.1	**18.0**
Academic stress	525	78.6	1.5	76.4	1.8	88.2	2.9	7.12	1	**0.007**	**2.1**	1.1	3.9	**39.6**
Sexual/Gender Identity Crisis	47	7.0	1.0	6.0	1.0	14.0	3.1	8.29	1	**0.004**	**3.3**	1.7	6.7	**7.6**
Hospitalization	65	9.8	1.1	9.2	1.2	14.6	3.2	2.83	1	0.092	1.7	0.9	3.2	4.7
You were involved in a life-threatening accident	28	4.2	0.8	4.2	0.8	4.1	1.9	0.01	1	0.941	1.1	0.4	3.1	0.2
You were seriously physically assaulted	25	3.7	0.7	3.3	0.8	4.5	2.1	0.38	1	0.569	2.2	0.7	6.3	2.0
You were sexually assaulted or raped	3	0.5	0.2	0.3	0.2	0.8	0.8	f	–	0.126	2.0	0.2	25.0	0.5
You had trouble with the police	14	2.2	0.6	2.2	0.6	1.1	1.0	0.52	–	0.497	1.1	0.2	6.5	0.2
You spent time in jail	2	0.4	0.2	0.4	0.3	–	–	f	–	1.000	1.7	0.0	62.7	0.1
Serious legal problem	9	1.4	0.5	1.5	0.5	–	–	f	–	0.612	0.4	0.0	8.7	−0.5
You had a pregnancy	2	0.3	0.2	–		1.6	1.1	f	–	**0.032**	14.5	0.3	699.7	1.0
You were diagnosed with a sexually transmitted infection	2	0.3	0.2	0.2	0.1	1.2	1.1	f	–	0.323	8.6	0.5	144.5	0.7
You were diagnosed with HIV	0	0.0	–	–	–	–	–	–	–	–	–	–	–	–
Any other recent experience	70	10.6	1.2	8.7	1.2	17.9	3.5	7.90	1	**0.005**	**2.4**	1.3	4.4	**8.8**
Any recent experience	552	90.7	1.2	89.2	1.4	96.1	1.8	4.34	1	**0.037**	2.4	0.9	6.6	49.6
No recent experience	57	9.3	1.2	10.8	1.4	3.9	1.8	–	–	–	(ref)	(ref)	(ref)	(ref)
Exactly one recent experience	133	21.9	1.6	23.5	1.8	13.1	3.0	–	–	–	1.2	0.4	3.9	2.4
Exactly two recent experiences	147	24.2	1.7	24.4	1.9	25.0	4.1	–	–	–	2.3	0.8	6.7	12.4
Three or more recent experiences	271	44.6	1.9	41.3	2.1	57.9	4.6	13.00	3	**0.004**	**3.1**	1.1	8.8	**35.1**

Significant *P*-values, OR and PARP are indicated in bold (α = 0.05); *OR* Odds ratio, *PARP* Population- attributable risk proportion

^a^The prevalence of the predictor in the full sample

^b^The prevalence of the predictor among those without 12-month depressive symptoms

^c^The prevalence of the predictor among those with 12-month depressive symptoms

^d^Unadjusted for multiple comparison. For multiple test adjusted *P*-values, see Additional file 1: Table S1

^e^Adjusted for gender and race

^f^Fisher-Exact test

**Table 4:** Multivariable Model adjusted for type and number of childhood-adolescent traumatic experiences

	MODEL 1					MODEL 2				
Predictor	OR	95%-	95%+	PARP	*P*-value^a^	OR	95%-	95%+	PARP	*P*-value^a^
A life-threatening illness or injury of a very close friend or family member	1.0	0.5	1.8	−0.6	0.913	0.9	0.5	1.7	−2.4	0.688
Death of a close friend or family member	0.7	0.4	1.3	−6.5	0.267	0.8	0.4	1.6	−3.2	0.588
Break-up with a romantic partner	1.0	0.6	1.8	0.2	0.974	1.1	0.6	2.0	1.7	0.819
You discovered that a romantic partner cheated on you	**2.1**	1.0	4.3	**7.7**	0.048	1.9	0.8	4.2	5.9	0.137
Serious betrayal by someone else close to you	1.2	0.7	2.2	4.2	0.499	0.9	0.5	1.7	−2.1	0.731
Serious ongoing arguments or break-ups with some other close friend or family member	**2.0**	1.1	3.6	**13.0**	0.023	1.7	0.9	3.3	9.2	0.113
Academic stress	1.5	0.8	3.1	24.3	0.235	1.3	0.6	2.8	14.3	0.493
Sexual/Gender Identity Crisis	**3.0**	1.3	6.8	**6.9**	0.008	2.0	0.7	5.2	3.4	0.168
Hospitalization	1.7	0.8	3.4	4.3	0.154	2.0	0.9	4.5	5.1	0.076
Any other stressful event	2.0	1.0	4.0	6.0	0.061	1.5	0.6	3.4	3.1	0.356
Exactly two recent stressful experiences	1.4	0.7	3.0	6.1	0.359	1.3	0.6	2.9	4.2	0.541
Three or more recent stressful experiences	1.0	0.4	2.9	0.3	0.987	0.8	0.3	2.6	−6.2	0.764
Parental psychopathology						**2.4**	1.2	4.8	**27.1**	0.016
Physical abuse						0.8	0.4	1.7	−3.8	0.502
Emotional abuse						**3.3**	1.5	7.1	31.2	0.003
Sexual abuse						0.8	0.2	2.8	−0.8	0.762
Neglect						**3.1**	1.4	7.1	**10.6**	0.006
Bullying victimization						**3.2**	1.6	6.2	**37.4**	0.001
Dating violence						**2.5**	1.1	5.5	**11.0**	0.028
Exactly two childhood adverse experiences						0.5	0.2	1.4	−10.3	0.194
Three or more childhood adverse experiences						0.3	0.1	1.3	−35.8	0.116

All analyses are adjusted for gender and race. Significant OR and PARP are indicated in bold (α = 0.05); OR = odds ratio; PARP = population- attributable risk proportion. Nagelkerke pseudo R-square for the first model was 9.48%; for the second model this was 15.02%. Area under the Curve for the first model was 0.722; for the second model this was 0.790

^a^unadjusted for multiple comparison. For multiple test adjusted P-values, see Additional file 1: Table S2

## Discussion

Our multivariable logistic regression model indicated that childhood adversity and three recent stressors were significantly associated with current depression in this cohort. Students reporting adverse events during childhood, which included neglect, emotional abuse and being bullied specifically, were about three times more likely to report current depression. From an individual- and population level perspective, emotional abuse and being bullied prior to the age of 17 were significantly associated to current depressive symptoms: The data indicate that roughly 32–38% of the reported depression in this cohort were attributable to these two types of adversities. With regard to recent stressors, the multivariable logistic regression model suggested that a romantic partner being unfaithful, serious ongoing arguments or break-ups with a close friend or family member and a sexual or gender identity crisis were the strongest predictors of depression during the past year.

These data are consistent with a broad range of studies indicating that experiences of childhood adversity like bullying [45] are associated with subsequent onset of psychopathology [46, 47]. Previous studies have also suggested that there is a dose-response relationship between childhood adversity and psychopathology [2, 48]. Similarly, the role of recent stressors in depression has been well explored in the literature [49, 50], with some evidence for the cumulative effect of childhood adversity and recent stressors [11, 51]. While several recent stressors have been found to be associated with depression, our adjusted multivariable model specifically found that gender identity crisis, a romantic partner being unfaithful and arguments within a family to be associated with depression. With regard to a gender identity crisis, a recent review by Turban and Ehrensaft [52] suggest that youth who experience gender identity crisis may be at risk of depression among other comorbid conditions. Similarly, terminating a romantic relationship has been found to be significantly linked to depression in several studies [53]. Reyes-Rodriguez and colleagues [54], who examined depression among a similar sample of college students in Puerto Rico, found that depression was associated with ending a romantic relationship [54]. Studies, albeit of adolescents and not college students, have suggested a plausible association between family arguments and depression [55, 56].

A number of important study limitations must be noted. First, the study was restricted to one university in South Africa, and we are not certain that the results do necessarily extrapolate to others. Second, a minority of the invitees completed the survey (12.9%). However, the use of sophisticated weighting techniques adjusted for the missing data. Third, our data are cross-sectional, and attempts to derive causal relationships are tentative. Fourth, despite our attempts to establish a closer temporal link between recent stressors and depression in the last year, we still cannot be entirely sure if the event (e.g. family arguments) preceded the onset of depressive symptoms. Family arguments could be an antecedent or a consequence of depression, as suggested by previous studies [57, 58]. Fifth, self-reports of childhood adversity may be subject to recall bias. Our survey was designed as a self-report and was completed without the assistance of an interviewer in an attempt to limit recall bias and to collect information that was as accurate as possible [59]. Sixth, our measures of childhood adversity and recent stressors may not however adequately capture the severity or duration of traumatic experiences [60]. Seventh, due to limited statistical power, we were unable to examine associations for sub-syndromal depression and major depressive disorder separately, and to include low-prevalent predictor variables in the final multivariate models. Larger sample sizes are needed to address these objectives in the future. Lastly, this is an exploratory study in which multiple analyses were undertaken without correction. Future confirmatory research should formally test the information this study generated. These phenomena deserve future study.

Despite these limitations, our findings suggest that a history of some childhood adverse events and recent stressors are associated with depression in students attending a tertiary institution for the first time. This association may be particularly pronounced during the transitional period of adolescence to early adulthood, characteristic of the university/college years. Further retrospective and prospective studies with larger samples are needed to examine the interaction between trauma, stress and depression.

## Conclusions

In conclusion, the college years are associated with increased risk of depression [61, 62]. Our findings suggest a significant relationship between early adversity, specific recent stressors, and depression in our student cohort, consistent with the broader literature on predictors of depression. Future research should address the impact of childhood trauma and recent stressors on wellbeing, quality of life, academic performance and choices after graduation. While this cross-sectional study underlines a number of variables that are associated with depression, longitudinal research is needed to fully delineate the relevant causal relationships. Universities could be natural sites of awareness campaigns of mental health related issues and intervention strategies and options, both at the prevention and treatment level [63]. First year students should be made aware of, and encouraged to make use of locally available mental health services for psycho-social support and to improve coping strategies.

## Additional file

Additional file 1: Table S1. Adjusted *P*-values after adjustment procedures for multiple comparison (Tables 2 & 3). **Table S2** Adjusted *P*-values after adjustment procedures for multiple comparison (Table 4). (DOCX 22 kb)

## Footnotes

1 The South African Apartheid system created population categories including White, Black African, Indian and Coloured. The term ‘Indian’ refers to individuals whose ancestors are from India. The term ‘Coloured’ broadly refers to individuals of mixed ancestry. These categories are still used in census and statistical data; we use them here not in order to reify these sociocultural categories, but rather because we are interested in exploring potential health disparities between population groups.
