# Supplementary material for: The relationship between childhood adversity, recent stressors, and depression in college students attending a South African university
Source: BMC Psychiatry. 2018 Mar 9;18:63. doi: 10.1186/s12888-017-1583-9 (PMC5845179; doi:10.1186/s12888-017-1583-9)
Supplement: Supplementary file 1 — Adjusted P-values after adjustment procedures for multiple comparison (Tables 2 & 3). Table S2 Adjusted P-values after adjustment procedures for multiple comparison (Table 4). (DOCX 22 kb) [file 12888_2017_1583_MOESM1_ESM.docx]

**Additional file 1: Table S1 Adjusted P-values after adjustment procedures for multiple comparison (Table 2 & 3).**

| Predictor | **RAW** | **BON** | **HOL** | **FDR** |
| --- | --- | --- | --- | --- |
| Parental psychopathology | **0.0024** | 0.0701 | **0.0402** | **0.0086** |
| Physical abuse | 0.1388 | 1.0000 | 0.8711 | 0.1917 |
| Emotional abuse | **0.0000** | **<.0001** | **<.0001** | **<.0001** |
| Sexual abuse | **0.0061** | 0.1774 | 0.0917 | **0.0127** |
| Neglect | **0.0008** | **0.0240** | **0.0182** | **0.0034** |
| Bullying victimization | **0.0000** | **<.0001** | **<.0001** | **<.0001** |
| Dating violence | **0.0045** | 0.1317 | 0.0681 | **0.0110** |
| Any adverse experience | **0.0003** | **0.0077** | **0.0063** | **0.0013** |
| Number of adverse experiences | **0.0000** | **<.0001** | **<.0001** | **<.0001** |
| A life-threatening illness or injury of a very close friend or family member | 0.6222 | 1.0000 | 1.0000 | 0.6683 |
| Death of a close friend or family member | 0.1648 | 1.0000 | 0.9333 | 0.2173 |
| Break-up with a romantic partner | 0.0743 | 1.0000 | 0.6685 | 0.1197 |
| You discovered that a romantic partner cheated on you | **0.0027** | 0.0771 | **0.0425** | **0.0086** |
| Serious betrayal by someone else close to you | **0.0002** | **0.0056** | **0.0047** | **0.0011** |
| Serious ongoing arguments or break-ups with some other close friend or family member | **0.0000** | **0.0005** | **0.0005** | **0.0001** |
| Academic stress | **0.0071** | 0.2057 | 0.1064 | **0.0137** |
| Sexual/Gender Identity Crisis | **0.0039** | 0.1128 | 0.0584 | **0.0103** |
| Hospitalization | 0.0920 | 1.0000 | 0.7418 | 0.1404 |
| You were involved in a life-threatening accident | 0.9409 | 1.0000 | 1.0000 | 0.9745 |
| You were seriously physically assaulted | 0.5693 | 1.0000 | 1.0000 | 0.6603 |
| You were sexually assaulted or raped | 0.1260 | 1.0000 | 0.8711 | 0.1826 |
| You had trouble with the police | 0.4971 | 1.0000 | 1.0000 | 0.6007 |
| You spent time in jail | 1.0000 | 1.0000 | 1.0000 | 1.0000 |
| Serious legal problem | 0.6124 | 1.0000 | 1.0000 | 0.6683 |
| You had a pregnancy | **0.0316** | 0.9163 | 0.3792 | 0.0573 |
| You were diagnosed with a sexually transmitted infection | 0.3226 | 1.0000 | 1.0000 | 0.4068 |
| You were diagnosed with HIV |  |  |  |  |
| Any other recent experience | **0.0050** | 0.1451 | 0.0751 | **0.0112** |
| Any recent experience | **0.0370** | 1.0000 | 0.4069 | 0.0631 |
| Number of recent experiences | **0.0039** | 0.1128 | 0.0583 | **0.0103** |

Note: RAW = unadjusted P-values as reported in Table 2-3; BON = Bonferroni adjustment; HOL = step-down Bonferroni method of Holm^1^; FDR = linear step-up method of Benjamini and Hochberg^2^. Significant P-values are indicated in bold (α=0.05).

1. Holm, S. (1979), “A Simple Sequentially Rejective Bonferroni Test Procedure,” Scandinavian Journal of Statistics, 6, 65–70.

2. Benjamini, Y. and Hochberg, Y. (1995), “Controlling the False Discovery Rate: A Practical and Powerful Approach to Multiple Testing,” Journal of the Royal Statistical Society, B, 57, 289–300.

**Additional file 1: Table S2 Adjusted P-values after adjustment procedures for multiple comparison (Table 4).**

|  | **MODEL 1** | | | | **MODEL 2** | | | |
| --- | --- | --- | --- | --- | --- | --- | --- | --- |
|  | **RAW** | **BON** | **HOL** | **FDR** | **RAW** | **BON** | **HOL** | **FDR** |
| A life-threatening illness or injury of a very close friend or family member | 0.9133 | 1.0000 | 0.9869 | 0.9869 | 0.6880 | 1.0000 | 0.8187 | 0.7962 |
| Death of a close friend or family member | 0.2669 | 1.0000 | 0.9869 | 0.4795 | 0.5876 | 1.0000 | 0.8187 | 0.7345 |
| Break-up with a romantic partner | 0.9738 | 1.0000 | 0.9869 | 0.9869 | 0.8187 | 1.0000 | 0.8187 | 0.8187 |
| You discovered that a romantic partner cheated on you | **0.0478** | 0.7656 | 0.5742 | 0.1914 | 0.1370 | 1.0000 | 0.8187 | 0.3426 |
| Serious betrayal by someone else close to you | 0.4989 | 1.0000 | 0.9869 | 0.6652 | 0.7312 | 1.0000 | 0.8187 | 0.7962 |
| Serious ongoing arguments or break-ups with some other close friend or family member | **0.0229** | 0.3668 | 0.2980 | 0.1223 | 0.1128 | 1.0000 | 0.8187 | 0.3216 |
| Academic stress | 0.2345 | 1.0000 | 0.9869 | 0.4795 | 0.4932 | 1.0000 | 0.8187 | 0.7345 |
| Sexual/Gender Identity Crisis | **0.0083** | 0.1322 | 0.1239 | 0.0661 | 0.1684 | 1.0000 | 0.8187 | 0.3828 |
| Hospitalization | 0.1535 | 1.0000 | 0.9869 | 0.4094 | 0.0761 | 1.0000 | 0.8187 | 0.2717 |
| Any other stressful event | 0.0611 | 0.9783 | 0.6574 | 0.1957 | 0.3557 | 1.0000 | 0.8187 | 0.6352 |
| exactly two recent stressful experiences | 0.3586 | 1.0000 | 0.9869 | 0.5215 | 0.5409 | 1.0000 | 0.8187 | 0.7345 |
| three or more recent stressful experiences | 0.9869 | 1.0000 | 0.9869 | 0.9869 | 0.7644 | 1.0000 | 0.8187 | 0.7962 |
| Parental psychopathology |  |  |  |  | **0.0161** | 0.4034 | 0.3227 | 0.0807 |
| Physical abuse |  |  |  |  | 0.5021 | 1.0000 | 0.8187 | 0.7345 |
| Emotional abuse |  |  |  |  | **0.0028** | 0.0694 | 0.0639 | **0.0231** |
| Sexual abuse |  |  |  |  | 0.7620 | 1.0000 | 0.8187 | 0.7962 |
| Neglect |  |  |  |  | **0.0061** | 0.1527 | 0.1344 | **0.0382** |
| Bullying victimization |  |  |  |  | **0.0007** | 0.0176 | 0.0168 | **0.0111** |
| Dating violence |  |  |  |  | **0.0285** | 0.7122 | 0.5481 | 0.1187 |
| exactly two childhood adverse experiences |  |  |  |  | 0.1942 | 1.0000 | 0.8187 | 0.4045 |
| three or more childhood adverse experiences |  |  |  |  | 0.1158 | 1.0000 | 0.8187 | 0.3216 |

Note: RAW = unadjusted P-values as reported in Table 4; BON = Bonferroni adjustment; HOL = step-down Bonferroni method of Holm^1^; FDR = linear step-up method of Benjamini and Hochberg^2^. Significant P-values are indicated in bold (α=0.05).

1. Holm, S. (1979), “A Simple Sequentially Rejective Bonferroni Test Procedure,” Scandinavian Journal of Statistics, 6, 65–70.

2. Benjamini, Y. and Hochberg, Y. (1995), “Controlling the False Discovery Rate: A Practical and Powerful Approach to Multiple Testing,” Journal of the Royal Statistical Society, B, 57, 289–300.
